# Global venomous snake redistribution and snakebite risk under climate change

**DOI:** 10.1093/cz/zoaf070

**Published:** 2025-11-06

**Authors:** Wei She, Xinru Wan, Weixiang Guan, Guangping Huang, Huizhong Fan, Ziyan Liao, He Zhang, Lei Luo, Fuwen Wei

**Affiliations:** Jiangxi Provincial Key Laboratory of Conservation Biology, College of Forestry, Jiangxi Agricultural University, No. 1101, Fangzhimin Avenue, Nanchang Economic and Technological Development Zone, Nanchang 330045, China; Jiangxi Provincial Key Laboratory of Conservation Biology, College of Forestry, Jiangxi Agricultural University, No. 1101, Fangzhimin Avenue, Nanchang Economic and Technological Development Zone, Nanchang 330045, China; Jiangxi Provincial Key Laboratory of Conservation Biology, College of Forestry, Jiangxi Agricultural University, No. 1101, Fangzhimin Avenue, Nanchang Economic and Technological Development Zone, Nanchang 330045, China; Jiangxi Provincial Key Laboratory of Conservation Biology, College of Forestry, Jiangxi Agricultural University, No. 1101, Fangzhimin Avenue, Nanchang Economic and Technological Development Zone, Nanchang 330045, China; State Key Laboratory of Animal Biodiversity Conservation and Integrated Pest Management, Institute of Zoology, Chinese Academy of Sciences, No.5, Yard No. 1, Beichen West Road, Beijing 100101, China; Chengdu Institute of Biology, Chinese Academy of Sciences, 23 Qunxian South Street, Chengdu 610041, China; Jiangxi Provincial Key Laboratory of Conservation Biology, College of Forestry, Jiangxi Agricultural University, No. 1101, Fangzhimin Avenue, Nanchang Economic and Technological Development Zone, Nanchang 330045, China; State Key Laboratory of Genetic Evolution and Animal Models, Kunming Institute of Zoology, Chinese Academy of Sciences, 17 Longxin Road, Kunming 650201, China; Jiangxi Provincial Key Laboratory of Conservation Biology, College of Forestry, Jiangxi Agricultural University, No. 1101, Fangzhimin Avenue, Nanchang Economic and Technological Development Zone, Nanchang 330045, China; State Key Laboratory of Animal Biodiversity Conservation and Integrated Pest Management, Institute of Zoology, Chinese Academy of Sciences, No.5, Yard No. 1, Beichen West Road, Beijing 100101, China

**Keywords:** biodiversity, climate change, distribution, public health, snake, venom

## Abstract

Climate change is reshaping the global distribution of venomous snakes, with important implications for public health. Snakebite envenoming is estimated to cause approximately 63,400 deaths annually, mostly in tropical and developing countries. However, few studies have comprehensively assessed the future global patterns of envenomation risk, particularly across venom types with differing clinical and ecological characteristics. In this study, we modeled the current and future distributions of 193 medically important venomous snake species (neurotoxic and hemotoxic) under 4 climate change scenarios through 2100. We assessed 3 indices to quantify snakebite risks: the Venom Richness Index (VRI) for species-specific venom diversity, the Snakebite Range Expansion Index (SREI) to capture range expansion or contraction, and the Venom Overlap Index (VOI) for geographic co-occurrence of venom types. We found that snakebite risk is projected to increase in temperate and cold regions under high-emissions scenarios, with VRI and SREI increasing in areas such as North America, Europe, and East Asia. In contrast, VRI and SREI are projected to decline across parts of tropical Africa, India, and southern China. VRI transitions are projected to expand in northern Mexico, Brazil, Spain, southwestern China, and southern Australia, increasing the potential for diagnostic uncertainty and treatment complexity. VOI is expected to rise in highly populated but poorly urbanized regions, highlighting the vulnerability of developing countries with limited infrastructure. These spatial and venom-specific shifts underscore the need for regionally tailored antivenom strategies, improved diagnostics, and high-resolution risk forecasting to mitigate the future burden of snakebite under global change.

## Graphical Abstract

**Graphical Abstract:**
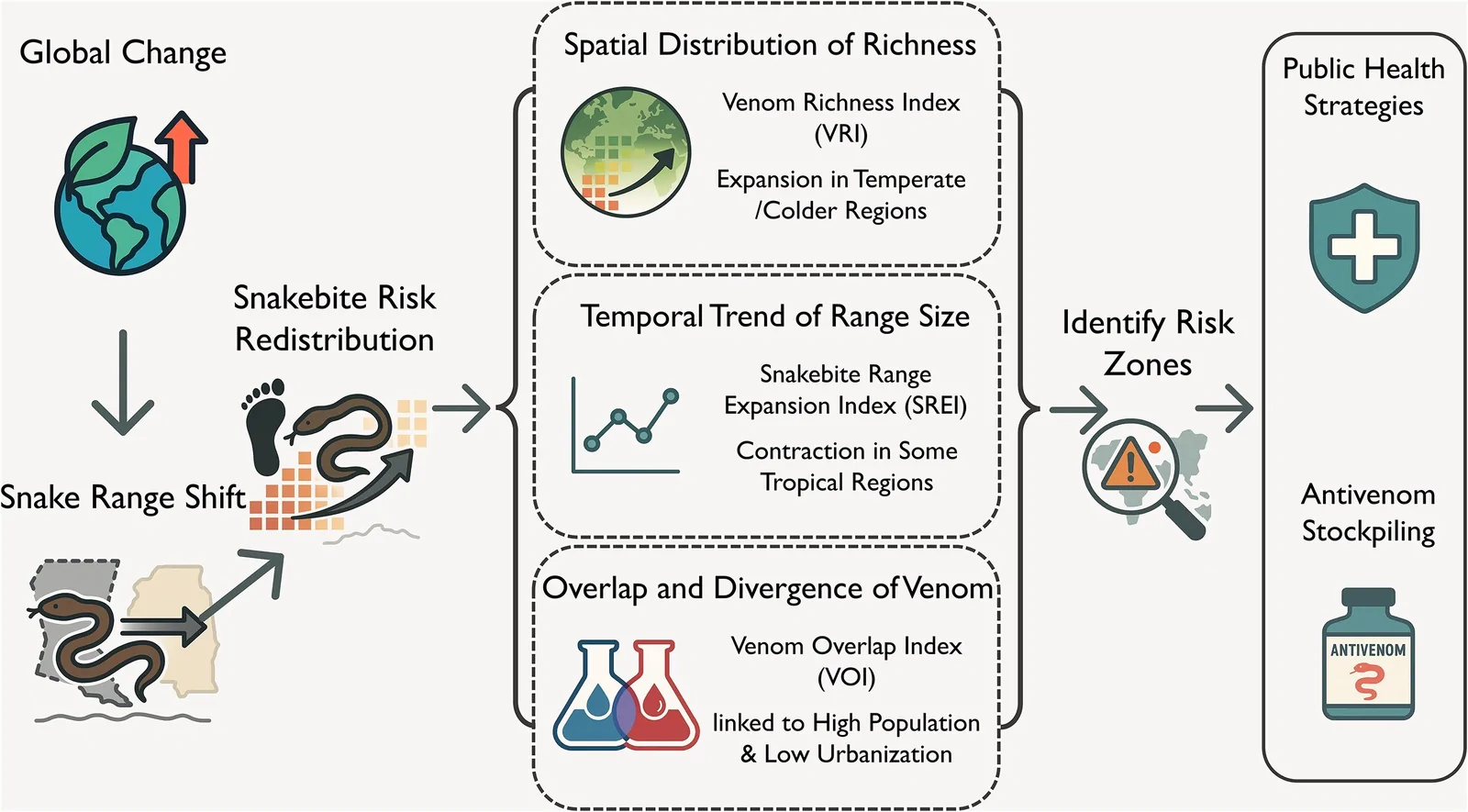

Climate change is having widespread and accelerating effects on global biodiversity; these effects are expected to intensify, leading to significant biodiversity loss and species range shifts (Wan et al. 2019). Global change-driven range shifts and biodiversity loss significantly aggravate ecological risks (Wan et al. 2022), increasing disease emergence, redistributing natural resources, as well as altering the composition of ecological communities (Pecl et al. 2017). Many venomous species, including snakes, are expanding into new areas under climate change, resulting in heightened spatial overlap with areas of human habitation in some areas. This biogeographic redistribution increases the frequency of human–wildlife encounters, thereby amplifying the risk of bites, envenomation, and associated public health threats (Martinez et al. 2022).

Snakebite envenoming is a severe and long-neglected global public health challenge, with an estimated 5 million bites occurring annually worldwide and resulting in approximately 63,400 deaths annually (38,900–78,600) (Reoberts et al. 2022). Considering its high morbidity and mortality, as well as the lack of effective prevention and treatment in endemic areas, the World Health Organization (WHO) reclassified snakebite as a Category A neglected tropical disease, indicating its high public health priority (Chippaux 2017). Therefore, snakebite is a significant health concern in areas where several venomous species exist. For example, in southern and eastern China, the Chinese cobra (*Naja atra*) is the most common venomous snake, particularly during summer, often affecting young adult males engaged in outdoor activities (Wang et al. 2014). The Chinese moccasin (*Deinagkistrodon acutus*), also found in these regions, poses additional risk due to its potent venom, which can cause coagulopathy and rapid fatality without prompt treatment (Huang et al. 2021). Venomous snakes such as the saw-scaled or carpet viper (*Echis ocellatus*), and to a lesser extent, African cobras (*Naja* spp.) and puff adders (*Bitis arietans*) account for the majority of severe envenomations and snakebite-related deaths in West Africa (Habib 2013). In South America, *Bothrops* species are the leading cause of snakebites, often resulting in coagulopathy and tissue damage (Nori et al. 2014). In Australia, snakes such as the taipan snake (*Oxyuranus scutellatus*) pose serious risks due to their potent neurotoxic venom, although fatalities are rare because of effective antivenom availability and strong healthcare systems (Herrera et al. 2012; Martinez et al. 2024). These regional examples underscore the urgent need for region-specific assessment of venomous snake distributions.

From a public health perspective, regional snakebite risk reflects a combination of species composition, ecological context, and human-driven changes such as habitat destruction, climate change, and urban expansion. Separately, the WHO also maintains a categorization of venomous snake species based on their medical importance. Currently, 130 species are listed as Category 1 (of highest medical importance) and 321 species as Category 2 (of moderate importance) (WHO 2024). Based on their primary toxicological effects, snake venoms are usually categorized into 2 functional types: neurotoxic venoms, which affect the nervous system and can cause respiratory paralysis (e.g., cobras, kraits, mambas); and hemotoxic venoms, which impair the circulatory system, leading to coagulopathy, hemorrhage, and tissue necrosis (e.g., Viperidae family). These 2 venom types lead to distinct clinical syndromes and require different antivenoms. In regions where snakes possess both venom types, rapid clinical diagnosis and appropriate treatment constitute a major challenge.

Driven by climate change and habitat transformation, the richness and geographic distribution of venomous snakes are undergoing significant shifts, with implications for envenomation risk. For example, in Mozambique, 9 of 13 venomous snake species are projected to lose suitable habitat, showing a northward shift that may alter public health vulnerability (Zacarias and Loyola 2019). In Argentina, climate models found moderate southward displacement of suitable niches for venomous snakes (Nori et al. 2014). In the United States, the range of the eastern coral snake (*Micrurus fulvius*) is shifting as the southeastern region moves beyond its historical climate envelope (Archis et al. 2018). Although many studies forecast a net contraction in suitable habitats, some areas, particularly those areas experiencing a particularly high human population growth, may face increased snake presence and associated envenomation risks (Martinez et al. 2024). Despite growing evidence of climate-driven range shifts, it remains unclear which venom types (i.e., neurotoxic or hemotoxic) will expand or emerge in specific regions, complicating public health planning and clinical response.

Therefore, high-resolution global forecasting is needed to capture not only the spatial dynamics of venomous snake species under climate change but also the potential disparity and overlap of different venom types. We hypothesize that: 1) neurotoxic and hemotoxic snake species may respond differently to climate change across biogeographic regions; 2) hot spots of neurotoxic–hemotoxic overlap are likely to increase in specific transitional regions, exacerbating diagnostic and treatment complexity; 3) snakebite risk may rise disproportionately in certain regions, such as those experiencing rapid population growth but limited urban development, thereby increasing public health vulnerability. To test these hypotheses, we modeled the global distributions of 193 medically important venomous snake species by integrating high-resolution climate and land-use projections with 3 complementary risk metrics: 1) type-specific species richness to identify ecological hot spots (Venom Richness Index, VRI); 2) temporal changes in the geographic coverage of high-risk areas to assess snakebite spread under climate change (Snakebite Range Expansion Index, SREI); 3) venom-type co-occurrence to highlight areas of clinical complexity (Venom Overlap Index, VOI). Using this integrated approach, we aimed to investigate the following scientific questions: 1) What are the current global patterns of venomous snake richness (as represented by VRI); 2) How will the geographic ranges and high-risk areas of these snakes evolve spatially and temporally under different future climate change scenarios? (as represented by VRI and SREI) 3) What are the key climatic, land-use, and anthropogenic factors shaping the spatial dynamics of snakebite risk (as represented by VOI)? Through a comprehensive analysis of these questions, this study aims to provide a robust recommendation for global public health policymakers and antivenom production and distribution agencies.

## Materials and methods

### Data collection and preparation

We compiled a list of 373 medically important venomous snake species based on WHO Category 1 and 2 classifications (WHO 2024), treating species assigned to both categories as Category 1 for consistency (Martinez et al. 2024) (Supplementary Table S1). Information on venom composition (neurotoxic, hemotoxic, or both) was retrieved for 304 of these species from the Clinical Toxinology Resources database (accessed June 2024). Occurrence records were compiled from the Global Biodiversity Information Facility (GBIF; 457,756 records) (GBIF.2024), iNaturalist (193,481 records), and The Atlas of Living Australia (43,112 records), resulting in an initial dataset of 694,349 records. To ensure spatial, temporal, and taxonomic accuracy, we applied a multistep filtering procedure to the raw occurrence dataset. Records lacking geographic coordinates (*n* = 4,369) or temporal information such as event date or year (*n* = 42,496), as well as those outside the 1900–2025 range (*n* = 1,764), were excluded. Only species- or subspecies-level records marked as “PRESENT” were retained. To reduce spatial redundancy, duplicate records for the same species within a 0.25° grid cell (roughly 27 km × 27 km at the equator) were excluded (Supplementary Figure S1A), to match the spatial resolution of the environmental data. This spatial filtering step yielded 56,567 distinct records of 293 snake species (Supplementary Figure S1B). Among these, 273 species had more than 5 valid distribution records and were included for subsequent species distribution modeling, a threshold commonly used to balance data quality with species representation (Supplementary Table S2). Although underreporting and data gaps are indeed global issues, Southeast Asia posed a particularly severe challenge in our dataset, with many medically important species entirely missing from occurrence databases (Supplementary Figure S1B). To avoid excluding ecologically and medically important Southeast Asian species from the modeling, we integrated expert-derived extant range maps from the IUCN Red List (IUCN 2024).

We selected 12 predictor variables to represent key ecological, climatic, and anthropogenic drivers of venomous snake distributions (Supplementary Table S3). Our selection process prioritized bioclimatic coverage by ensuring the inclusion of representative variables for both temperature (mean annual temperature) and precipitation (annual precipitation), as these are foundational determinants of ectothermic physiology and distribution (Fick and Hijmans 2017). We then screened additional candidate variables based on the variance inflation factor (VIF < 5) to reduce multicollinearity (O’Brien 2007), while maintaining ecological relevance. Land-use categories, including primary forest, secondary forest, rangeland, pasture, primary non-forest, secondary non-forest, and urban, were treated as independent continuous predictors (fractional cover), representing habitat quality and human disturbance (Chini et al. 2014). We also included human population density as a proxy for human disturbance (Gao 2017), and elevation to capture altitudinal gradients and topographic constraints (Fick and Hijmans 2017). To assess regional differences in species’ environmental responses, grid cells were classified into climatic zones (tropical, arid, temperate, and cold–polar category) based on the Köppen–Geiger system, which defines global climates according to temperature and precipitation patterns (Beck et al. 2023).

### Species distribution modeling

Species distribution modeling was carried out using the Maxent algorithm (Li et al. 2024), a well-established machine learning approach known for its effectiveness with presence-only datasets such as those derived from GBIF (Hijmans et al. 2023; Urbanek 2024). Each species’ occurrence records were paired with the selected environmental predictors to estimate habitat suitability under current climatic conditions and future climate scenarios. Future projections were made across 4 Shared Socioeconomic Pathways (SSP126, SSP245, SSP370, SSP585) and 4 time intervals (2021–2040, 2041–2060, 2061–2080, and 2081–2100), enabling us to assess climate-driven shifts in species ranges across multiple scenarios (Supplementary Figure S2). Compared with other projections, results for SSP585 in the late 21st century should be interpreted with caution due to the potential over-extrapolation. To avoid over-projection, models were spatially constrained to the continent or island where each species currently occurs. In Southeast Asia, where occurrence data were sparse (Supplementary FigureS1B), we generated 1,000 simulated presence points within IUCN-defined extant ranges to better define spatial constraints. These points were not used for model training; the sensitivity analysis showed that including these points had a minor impact on predictor estimates (Supplementary Table S4).

To minimize spatial sampling bias at distributional edges, 10,000 pseudo-absence points were sampled from a 500 km buffered polygon around occurrence records. Ensemble model performance was evaluated using a sequential selection procedure, retaining models with the lowest average 10th percentile omission rates based on cross-validation. Model tuning involved testing linear, quadratic, and hinge feature classes, and the optimal model was identified based on the lowest Akaike Information Criterion (AIC) value (Muscarella et al. 2014). Species with sufficient occurrence data and low collinearity among predictors (*n* = 193) were retained for final modeling. These included 73 neurotoxic, 82 hemotoxic, and 17 mixed-type species, spanning both WHO Category 1 and 2 snakes (Supplementary Tables S5–S7). Projections incorporated outputs from 10 global climate models provided by the WorldClim dataset because of their complete bias-corrected coverage across all 4 SSPs and time periods, as well as their ensemble diversity to capture uncertainties: ACCESS-CM2, CMCC-ESM2, EC-Earth3-Veg, GISS-E2–1-G, INM-CM5-0, IPSL-CM6A-LR, MIROC6, MPI-ESM1-2-HR, MRI-ESM2-0, and UKESM1-0-LL (Fick and Hijmans 2017). To convert Maxent's continuous habitat suitability outputs into binary presence–absence maps, we applied the 10th percentile training presence threshold, which is commonly used to reduce noise from uncertain or ecologically suboptimal occurrences (Elith et al. 2011).

### Modeling spatial and temporal dynamics of snakebite risk

Snakebite risk was assessed using 3 metrics that capture ecological, clinical, and demographic dimensions of snakebite risk. First, we defined a VRI by identifying high-risk grid cells (0.25° × 0.25°, approximately 27 km × 27 km) containing 5 or more venomous snake species of the same venom type (either neurotoxic or hemotoxic). This threshold was selected to minimize the influence of isolated species occurrences and better capture areas with consistently high envenomation potential. To evaluate the robustness of this threshold, we tested alternative thresholds (e.g., 6–12 species), which yielded similar spatial patterns (Supplementary Figure S3), supporting the stability of our results.

Second, to evaluate how climate change may alter the geographic range of snakebite risk, we modeled SREI to quantify temporal changes in the geographic coverage of high-risk areas (Snakebite Range, SR). We modeled SREI using generalized linear mixed models (GLMMs) with a negative binomial distribution to account for overdispersion in count data (Brooks et al. 2017). The model was fitted using the formulas:


$$SR_{ijk}∼\mathrm{NB}(\mu_{ijk})$$

$$\begin{array}{r} \log\;(\mu_{ijk})=\beta_{0}+\beta_{1}\times \mathrm{Perio}d_{i}+\gamma_{j}^{\mathrm{Region}}+\delta_{k}^{\mathrm{SSP}}+\theta_{ij}^{\mathrm{Period}\times \mathrm{Region}}+u_{ijk} \end{array}$$

where the number of grid cells with VRI greater than 5 ($SR_{ijk}$) under different time periods (*i*), climate regions (*j*), and SSP scenarios (*k*) using a generalized linear mixed model with a negative binomial distribution (nbinom2) and a log link to account for overdispersed count data. $\mu_{ijk}$ is the expected number of occupied grid cells, $\beta_{0}$ is the intercept, $\beta_{1}$ captures the SREI as a primary slope, indicating the overall temporal trend in range size, $\gamma_{j}^{Region}$ and $\delta_{k}^{SSP}$ represent the fixed effects for climate zones and Shared Socioeconomic Pathways, respectively, $\theta_{ij}^{Period\times Region}$ accounts for regional-specific deviation from global temporal trend, $u_{ijk}$ is a random intercept for each climate model, capturing inter-model uncertainty.

Third, we calculated a VOI by multiplying the number of neurotoxic and hemotoxic species co-occurring within each cell, to highlight regions where divergent venom types may complicate clinical diagnosis and antivenom selection. To avoid zero values and retain the contribution of single-venom-type regions, one was added to each species count prior to multiplication. To model the environmental and anthropogenic drivers of VOI, we applied the Integrated Nested Laplace Approximation (INLA) framework with a Stochastic Partial Differential Equation (SPDE) approach (Rue et al. 2009). This spatially explicit hierarchical model incorporated a spatial random field with a Matérn covariance structure and was constructed over a triangulated mesh to account for spatial autocorrelation. The model was fitted using the formulas:


$$\mathrm{VO}I_{S}∼N(\mu_{S},\sigma^{2})$$

$$\mu_{S}=\beta_{0}+\overset{12}{\underset{J=1}{\sum }}\;\beta_{j}\times X_{\;js}+f(s)$$

where $\mathrm{VO}I_{S}$ is the venom-type overlap index at spatial location *S*, which is assumed to follow a normal distribution with expected mean of VOI ($\mu_{S}$) and variance $\sigma^{2}$. This is suitable for modeling a continuous, approximately Gaussian outcome. $\beta_{0}$ is the intercept, $X_{\;js}$ are the standardized covariates (e.g., temperature, precipitation, land-use, urban cover, population density, altitude), $\beta_{j}$ are the corresponding coefficients, and f(s) is the spatial random effect modeled using SPDE. There were 3 climate variables (mean temperature, precipitation, and temperature seasonality), 6 land-use types (primary forest, secondary forest, pasture, rangeland, primary non-forest, and secondary non-forest), 2 human-related factors (population density and urban land cover), and altitude in this analysis. Model performance was evaluated through 10-fold cross-validation, with spatial models consistently outperforming non-spatial alternatives in both predictive accuracy and model fit (lower DIC/WAIC). To estimate the influence of each predictor, we computed posterior distributions of effect sizes within the INLA-SPDE. GLMM models were implemented using the glmmTMB (Brooks et al. 2017), lmerTest (Kuznetsova et al. 2017), and car packages (Fox and Weisberg 2019). Species distribution models were carried out via the ENMeval package (Muscarella et al. 2014). INLA models were carried out via the INLA package (v24.12.11) (Rue et al. 2009). All statistical analyses were conducted in R (v4.5.0) (R Core Team 2025).

## Results

### Global pattern of venomous snake diversity and snakebite risk area

**Figure 1:**
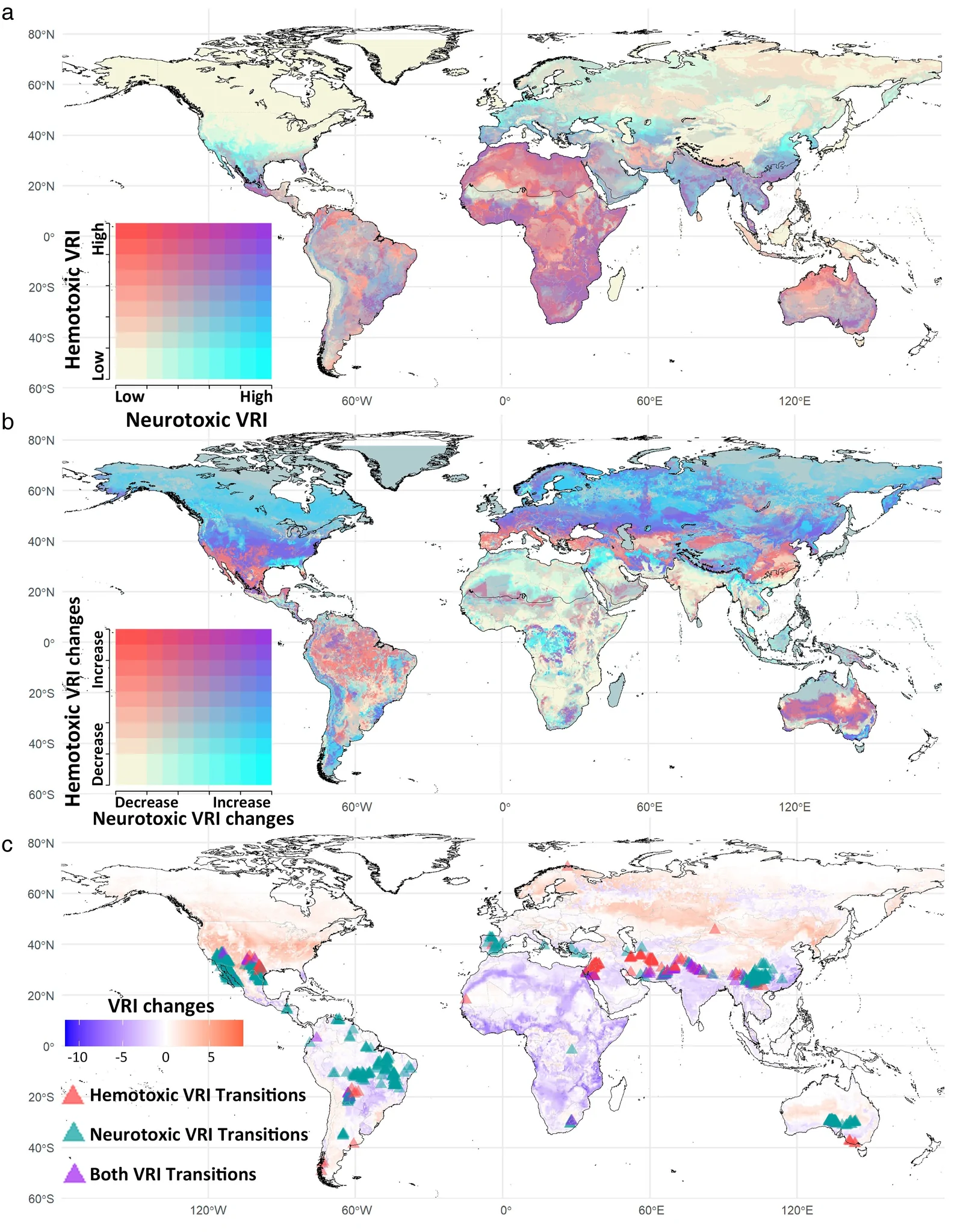
Current global patterns (a), projected changes (b) in neurotoxic and hemotoxic VRI from 2021 to 2100, and VRI transitions (c). (a and b) Background color represents the combined levels of VRI or VRI changes of neurotoxic (*x*-axis) and hemotoxic (*y*-axis) snake species, with purple colors indicating higher values of both VRI; both variables were linearly rescaled from 0 to 1. C: The background colors represent VRI changes for all snakes, triangle symbols denote grid cells where VRI transitions from <5 to >5 in neurotoxic (green), hemotoxic (red), or both venom types (purple). Map created using R version 4.5.0 and Adobe Photoshop 25.9.0. The Basemap was downloaded from the National Geomatics Center of China (https://www.ngcc.cn/).

We modeled the distributions of 193 medically important snake species from 4 families: Viperidae (99 species), Elapidae (84), Colubridae (5), and Atractaspididae (5) (Supplementary Table S7). These species span 44 genera and include 73 primarily neurotoxic, 82 hemotoxic, 17 dual-venom, and 55 unclassified species, providing a broad taxonomic and toxin-based representation of global snakebite risk. We identified region-specific patterns in the VRI for neurotoxic and hemotoxic species, with overlap hot spots (where both venom types co-occur) were found in West Africa, the eastern coast of Africa, Southeast Asia, and southern China (Fig. 1a). In contrast, higher hemotoxic than neurotoxic VRI were found in the Amazon Basin, whereas neurotoxic VRI was higher in the southern United States, Western Europe, and northern China (Fig. 1a).

Under future climate change, regions of higher neurotoxic and hemotoxic VRI were projected to expand into temperate, arid, and high-elevation regions (Fig. 1b). This expansion could lead to a significant increase in venom-type overlap, particularly in temperate zones of North America, Europe, and Asia. Meanwhile, both neurotoxic and hemotoxic VRI were expected to decline in parts of Africa, India, and southern China (Fig. 1b). Notably, hemotoxic VRI was projected to increase in Mexico and the Amazon Basin, whereas neurotoxic VRI increases in the Congo Basin but declines elsewhere in Africa (Fig. 1b).

Transitions from <5 to >5 venomous species (i.e., VRI threshold increases) were found in northern Mexico, Brazil, Spain, southwestern China, and southern Australia (Fig. 1c). Hemotoxic VRI increases were particularly evident in the Middle East, where the hemotoxic VRI is projected to rise. In contrast, regions experiencing simultaneous increases in both neurotoxic and hemotoxic VRI were concentrated in the mountainous areas of northern India and southwestern China (Fig. 1c), where overall VRI is expected to remain stable or increase, indicating heightened co-occurrence and potential clinical complexity.

### Spatial–temporal changes of snakebite risk areas under climate change

**Figure 2:**
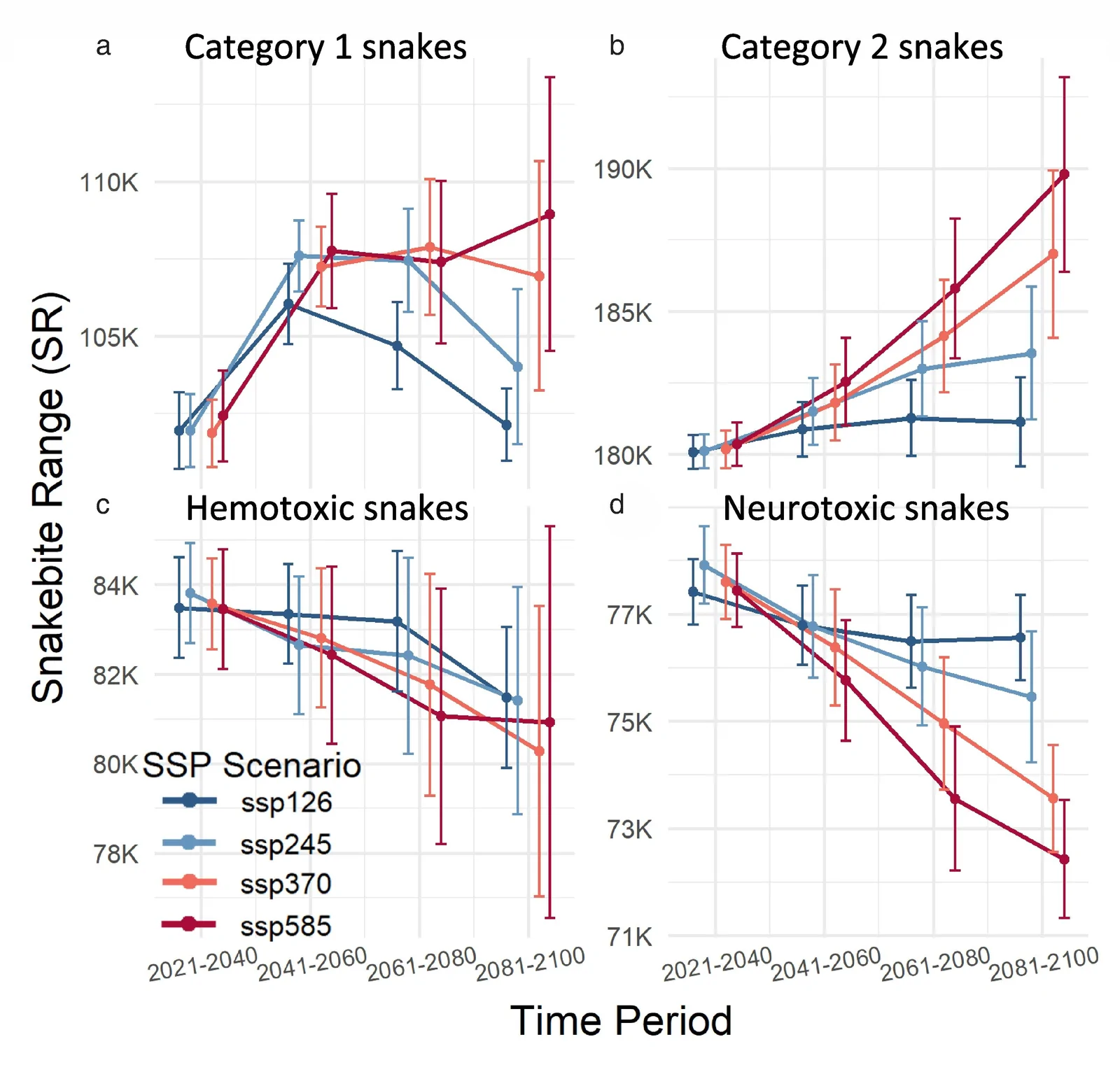
Temporal changes in the SR for Category 1 (a), Category 2 (b), hemotoxic (c), and neurotoxic (d) snake species under climate change scenarios. The SR was measured by the number of grids with VRI greater than 5. Lines and points represent the mean number of grids across 10 climate models, with error bars indicating standard deviation. Region-specific trends are represented in Supplementary Figure S4.

Projected climate warming was expected to drive sustained expansion in the SR (as measured by the number of grids with VRI greater than 5) for WHO Category 1 and 2 snakes (Fig. 2a and b). Under high-emissions scenarios (e.g., SSP585), the SR was projected to steadily increase through 2100, in contrast, the SR was expected to remain stable under low-emission scenarios (e.g., SSP126). The SR of hemotoxic snakes showed non-significant changes across all climate pathways (Fig. 2c), whereas SR of neurotoxic snakes showed a slight decline under high-emissions scenarios (Fig. 2d). The expansion or contraction of SR was found to vary across biogeographic regions (Supplementary Figure S4). In tropical regions, SR for all snake groups was projected to contract (Supplementary Figure S4A, E, I and M). In contrast, SR exhibited consistent expansion in cold regions, especially under SSP585 (Supplementary Figure S4D, H, L and P). Temperate and arid regions showed modest increase in SR for Category 1 and 2 snakes (Supplementary Figure S4B, C, F and G), whereas hemotoxic and neurotoxic species showed SR contractions in arid zones (Supplementary Figure S4J and N).

**Figure 3:**
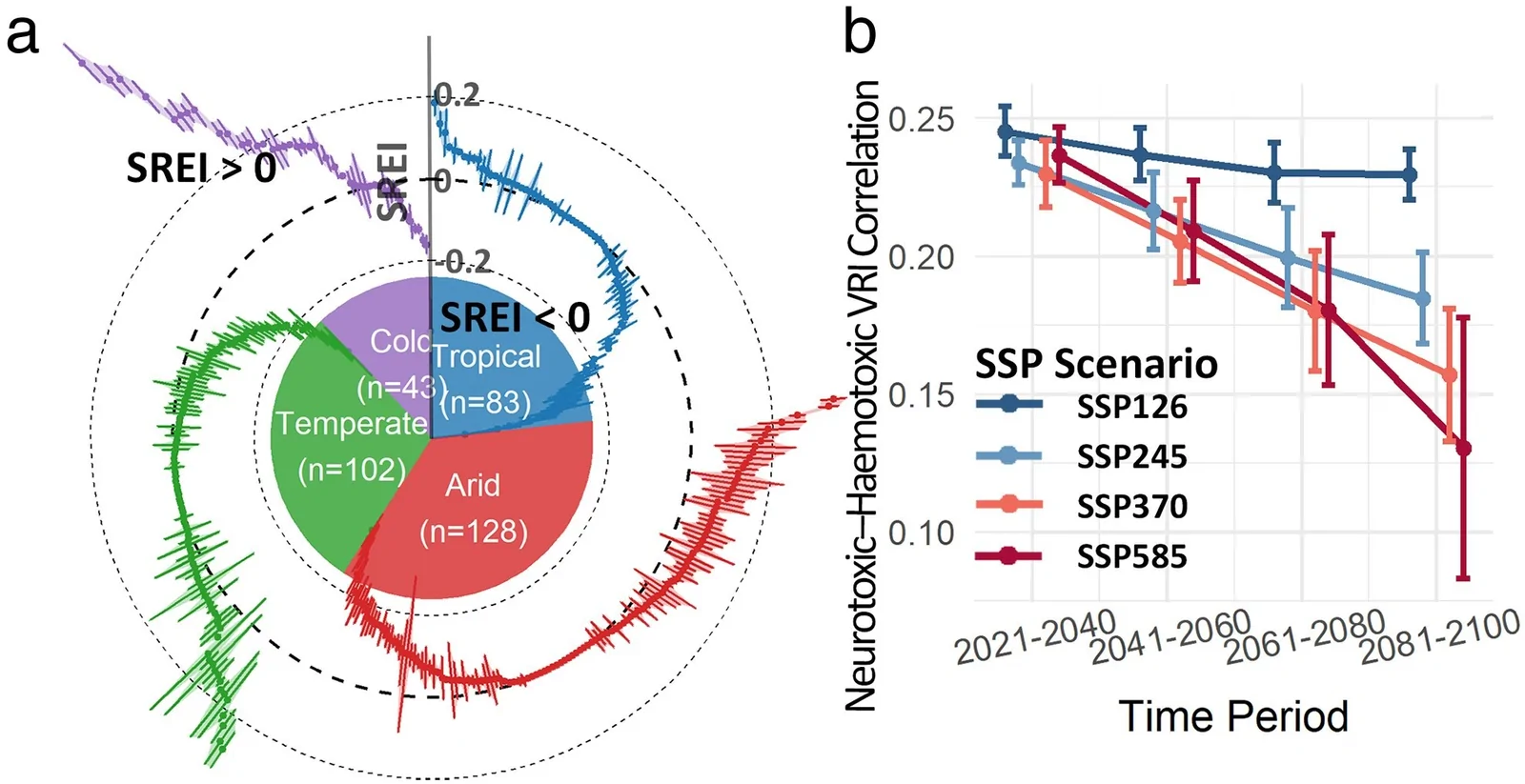
SREI across tropical, arid, temperate, and cold regions (a) and temporal trend in the correlations between neurotoxic VRI and hemotoxic VRI (b) under climate change. (a) Points represent SREI, with 95% confidence intervals (CI). Points outside the dashed zero line indicate range expansion, whereas points inside indicate contraction. Colors represent different regions, and the inset pie chart denotes the number of species per region, a summary table of SREI by region is provided in Table 1. (b) Spearman's correlation coefficients were averaged across climate models per scenario and time period, with error bars showing standard deviation. Region-specific trends are shown in Supplementary Figures S5 and S6.

At the species level, SREI further revealed that 110 species were projected to expand their ranges, whereas 113 species were expected to contract across different regions (Fig. 3a, Table 1). Changes in SREI were more closely associated with regional climate context than venom type: range expansions (reflected by positive SREI values) were more prevalent among species in cold regions (24 of 41 species), whereas more range contractions (as indicated by negative SREI) were more frequent among tropical species (35 of 75 species). Arid and temperate species were evenly divided between range expansion and contraction (Fig. 3a, Table 1). Notably, neurotoxic and hemotoxic snakes exhibited similar SREI patterns within each region (Supplementary Figure S5), suggesting that interspecific ecological traits and biogeography, rather than venom type, primarily mediated range shifts.

**Table 1:** The number of species exhibiting SR expansion or contraction across biogeographic regions under climate change

Region	Significant increase (SREI> 0)	Non-significant (CI overlaps 0)	Significant decrease (SREI < 0)	Total
Tropical	19	21	35	75
Arid	45	34	40	119
Temperate	22	42	30	94
Cold	24	9	8	41
Total	110	106	113	329

Note: Species totals exceed 193 due to some species distributed in multiple regions.

The SR expansion or contraction was also represented by the SREI with 95% CI in Fig. 3A.

### Human snakebite exposure and exposure factors

We observed a relatively low correlation (approximately 0.25) between hemotoxic and neurotoxic VRI, indicating some degree of co-occurrence. However, this correlation declines further under future warming scenarios (Fig. 3b), suggesting an increasing spatial segregation between the 2 venom types—i.e., regions may become dominated by one venom type rather than both, which could reduce co-occurrence even if statistical correlation persists. This decoupling was particularly pronounced in tropical and arid regions, whereas cold regions exhibited increasing correlations over time (Supplementary Figure S6). These patterns suggested that neurotoxic and hemotoxic snake distributions may become increasingly spatially segregated under climate change, potentially increasing the variability of venom risks according to regions.

**Figure 4:**
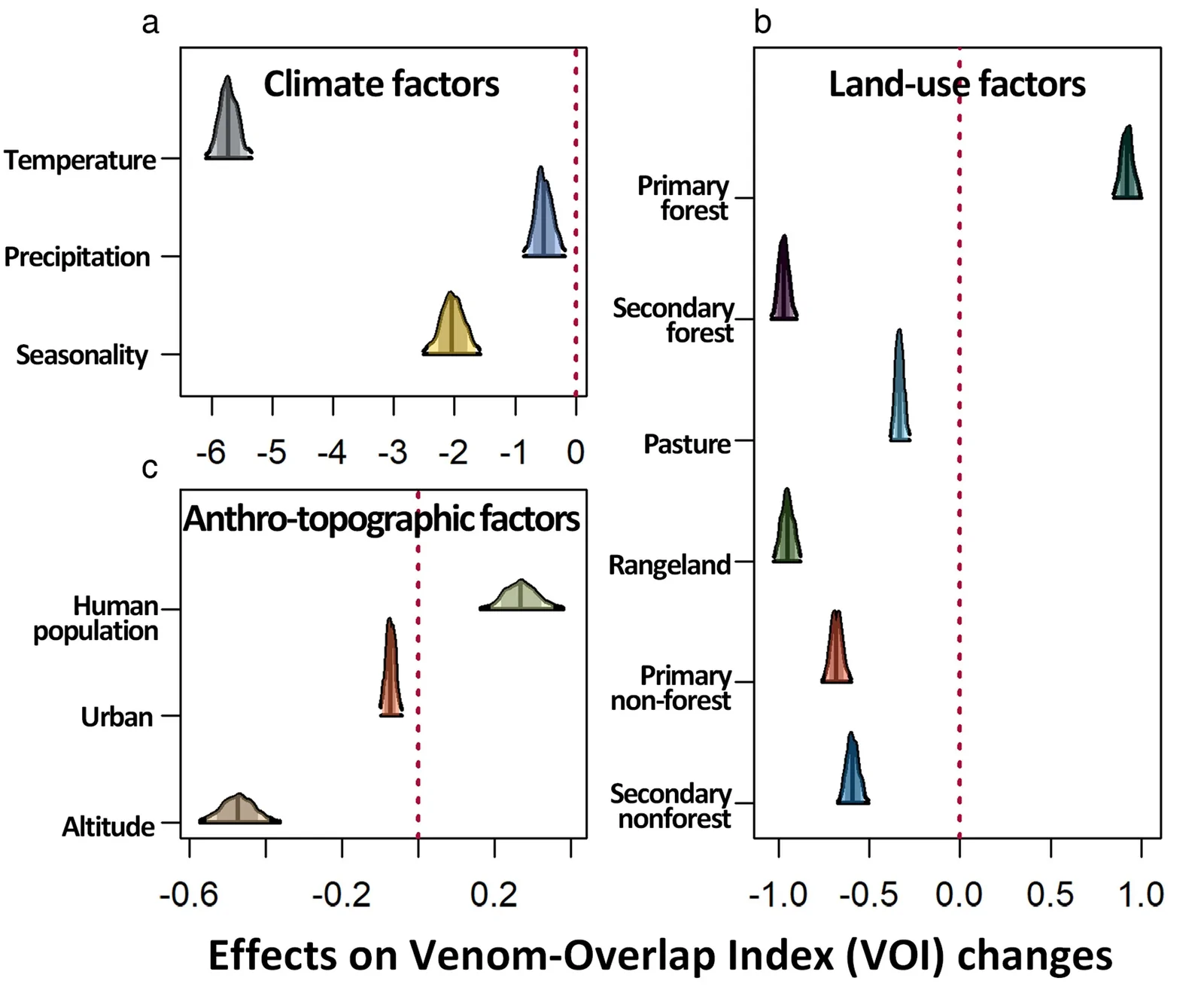
The effects of climatic (A), land-use (B), anthropogenic, and topographic (C) factors on VOI changes. The magnitude and direction of each predictor's effect are visualized as posterior density plots, with the 80% (darker shade) and 95% (lighter shade) credible intervals. The red dashed vertical line indicates a null effect (zero). An effect is statistically significant when its 95% credible interval does not cross this line. Distributions to the right of the zero line indicate a positive association (increased risk), whereas those to the left indicate a negative association (decreased risk).

Several key environmental and anthropogenic factors influencing changes in VOI were identified using a spatial Bayesian model (INLA-SPDE) (Fig. 4). Among climatic variables, higher mean annual temperature, precipitation, and temperature seasonality were significantly associated with lower VOI values (Fig. 4a). Regarding land use, higher coverage of primary forest positively correlated with increased VOI, whereas rangeland and pastures correlated with reduced VOI (Fig. 4b). Although urban expansion showed a slight negative effect on VOI, areas with high population density and low elevation were projected to face elevated VOI levels (Fig. 4c).

## Discussion

This study provides the first global, venom-type-specific assessment of snakebite risk under future climate change scenarios. Our findings demonstrate that climate change is driving significant shifts in the geographic distribution of venomous snakes, with snakebite risk (as indicated by VOI and SREI) projected to increase in temperate and cold regions whereas contracting in parts of the tropics. These patterns are shaped more by biogeographic context than by venom type, although the convergence of neurotoxic and hemotoxic VRI in certain mountainous regions poses significant clinical challenges. Conversely, the divergence of venom types in tropical and arid areas introduces new uncertainties for antivenom planning and species identification. We also found that snakebite risk (as indicated by VOI) is expected to rise in high-population but poorly urbanized regions, underscoring the vulnerability of many developing countries with limited diagnostic and treatment infrastructure. Although temporary declines in snake diversity may simplify clinical management in some areas, long-term shifts in snake assemblages and venom profiles are likely to complicate public health responses. These findings highlight the importance of biogeographic context over venom type in shaping future risk patterns.

### Global snakebite risk patterns and environmental drivers

Snakebite envenoming causes an estimated 63,400 deaths annually (38,900–78,600) and results in 2.94 million years of life lost (1.79–3.74 million). The highest burden is in South Asia, Southeast Asia, and sub-Saharan Africa, with Asia reporting the highest incidence and mortality rates. Europe has the lowest rates globally (Roberts et al. 2022). Previous studies found that snakebite risk is closely associated with climatic conditions, land use patterns, and human activity (Shashar et al. 2018; Landry et al. 2023). For example, higher temperatures and lower humidity are associated with increased snakebite risk in some semiarid nontropical areas (Shashar et al. 2018), likely due to increased snake mobility and greater human exposure during outdoor activities. In some areas in the southern United States, a 1 °C increase in daily maximum temperature has been linked to a 5–6% rise in the likelihood of snakebite incidents (Landry et al. 2023). Moreover, the presence of native or planted forests is positively correlated with snakebite risk, whereas intensively cultivated agricultural areas and urban environments tend to have lower risk levels (Collinson et al. 2025). Urbanization and higher population density are associated with a reduction in snakebite incidence globally, likely due to habitat fragmentation and fewer human–snake interactions in densely developed regions (Martín et al. 2024).

Corroborating with previous studies, this study indicates that climate change exerts a dual and regionally contrasting influence on the distribution of venomous snakes. Snakebite hot spots and range expansion indicators such as the VRI and SREI are projected to increase in cold and temperate regions, including parts of North America, Europe, and northern Asia (Fig. 1b, Table 1, Supplementary Figure S4,). Similar to patterns observed in other taxa such as birds, this expansion is primarily driven by rising temperatures, which reduce physiological thermal constraints for cold-sensitive species and facilitate both poleward and elevational range shifts (Jin et al. 2025). These results align with broader ecological evidence that ectothermic organisms are tracking shifting thermal niches in response to global warming (Simon and Amarasekare 2024). In contrast, snake species in tropical regions are expected to undergo contractions in suitable habitat. This decline is likely attributed to a combination of excessive rainfall, habitat degradation from deforestation, and intensified land-use pressures. Current studies have shown that snake species richness, abundance, and functional diversity decrease significantly in response to forest loss and habitat fragmentation. Landscapes characterized by high forest cover and contiguous patches support greater snake diversity, whereas fragmented or degraded habitats are associated with substantial declines in these ecological metrics (Leal-Santos et al. 2024).

Despite the distinct physiological effects of neurotoxic and hemotoxic venoms (Suhita et al. 2022), this study suggests that venomous snake species from both groups exhibit broadly similar geographic range shifts in response to climate change. This indicates that shared ecological constraints are likely the primary drivers of distributional changes. One plausible explanation is ecological niche breadth: species with wider environmental tolerances and greater dispersal capacities are more capable of expanding into novel habitats under changing climate conditions (Travis et al. 2013). These traits, commonly found across both venom types, enhance adaptive potential in response to environmental shifts (Wan et al. 2025; Wan and Wei 2025). Our findings show that range expansion in venomous snakes (as measured by SREI) is more strongly shaped by biogeographic context than venom type. Although climate is a key driver, its effects vary across biogeographic regions—cold-adapted species respond more strongly to warming and drying trends, whereas tropical species are more influenced by shifts toward cooler, wetter conditions. A species’ historical distribution further mediates its sensitivity to climate, emphasizing that regional ecological conditions are more predictive of future range dynamics than toxicological classification (Pacifici et al. 2020).

### Neurotoxic–hemotoxic co-distribution and divergence

Although neurotoxic venoms are commonly associated with Elapidae species (e.g., cobras, kraits), and hemotoxic effects are more prevalent among Viperidae (e.g., vipers, rattlesnakes) (Osipov and Utkin 2023), this relationship is not exclusive. Several Viperidae species, such as *Crotalus durissus* in South America, exhibit neurotoxic effects, whereas some Colubridae species can also deliver hemotoxic venom. In our dataset of 193 medically important species, 17 species were found to possess both hemotoxic and neurotoxic venom components, indicating overlapping venom phenotypes across families. Together, neurotoxic and hemotoxic venoms account for a substantial proportion of snakebite-related morbidity and mortality worldwide—38% and 25%, respectively (Suhita et al. 2022). Hemotoxic envenomations are also clinically significant, often causing coagulopathy, hemorrhage, necrosis, and renal complications, thus requiring targeted management and antivenom selection. Understanding the geographic distribution of venomous snake species and the prevalence of their associated venom types is essential for effective clinical management and targeted antivenom development, particularly in regions with high snakebite incidence.

Globally, the burden of snakebites is highest in tropical regions of sub-Saharan Africa, South Asia, and Southern America, where limited access to healthcare and antivenom further exacerbates envenomation risks (Kasturiratne et al. 2008; Chaisakul et al. 2020). Regional studies reveal substantial geographic variation in venom type prevalence. For instance, neurotoxic envenomation is more common in Africa, primarily due to the presence of Elapidae species, whereas hemotoxic snakebites are more frequently reported in parts of South India (Alfa-Ibrahim Adio et al. 2024). Moreover, both venom types can co-occur within the same country or region. In Ghana, for example, Elapid and Viperid species are distributed across all agro-ecological zones, with Elapids contributing to neurotoxic cases and Viperids dominating hemotoxic envenomations (Deikumah et al. 2023).

Our study identifies a concerning increase in the geographic overlap between neurotoxic and hemotoxic VRI, particularly in mountainous regions of South Asia (e.g., northern India and southwestern China) and East Africa. This spatial convergence poses significant clinical challenges. Higher VOI represents that frontline clinicians may encounter a broader variety of venom effects, necessitating rapid discrimination between neurotoxic and hemotoxic syndromes. This complexity elevates the need for polyvalent antivenoms that can neutralize multiple venom types simultaneously. In these regions, individuals may be bitten by snakes with fundamentally different venom mechanisms, increasing the risk of misdiagnosis and inappropriate treatment. Health care providers must differentiate between neurotoxic manifestations, such as respiratory paralysis, and hemotoxic effects, including coagulopathy and internal bleeding—often in settings with limited diagnostic infrastructure. This complexity highlights the urgent need for the development and deployment of polyvalent antivenoms capable of neutralizing multiple venom types (Chaisakul et al. 2020). Currently available antivenoms are typically region-specific and may not be effective in areas where venom types co-occur. Therefore, emerging zones of venom-type overlap should be prioritized for investment in polyvalent antivenom research and production, as well as for enhanced clinical training to improve treatment outcomes and reduce mortality.

In contrast to overlap zones, our analysis reveals growing spatial divergence between neurotoxic and hemotoxic VRI in tropical and arid regions. This “decoupling” suggests that some areas may become dominated by a single venom type, potentially simplifying treatment locally but increasing clinical uncertainty at broader scales. For health systems, this presents a major challenge, as shifting venom distributions complicate antivenom planning and species identification—especially in rural areas where diagnostic tools are limited and reliance on visual identification (Chaisakul et al. 2020). Thus, public health preparedness should remain flexible and region-specific. Antivenom deployment strategies, clinical protocols, and frontline training should be regularly updated to reflect evolving venom risks. Establishing real-time monitoring systems that integrate environmental change and snakebite incidence would be essential to reducing diagnostic uncertainty and improving outcomes, such as faster identification of venom types, timely antivenom administration, and reduced morbidity and mortality.

### Implications for prevention strategies

Compared with recent global assessments such as Martinez et al. (2024), which used broad-scale species presence and generalized environmental correlates to project snakebite risk, our study introduces a more ecologically detailed and venom-type-specific approach. Rather than assuming uniform toxicity across taxa, we distinguish neurotoxic and hemotoxic species, enabling spatial projections of venom-specific risk. We introduce the VRI, VOI, and SREI to quantify projected range shifts of medically important species across biogeographic regions, these metrics provide a more mechanistic and spatially resolved understanding of how snake assemblages and venom risks are likely to change over time. This approach complements existing models by emphasizing ecological traits and venom diversity, which are important for guiding region-specific antivenom development and public health strategies.

A key finding of this study is that projected increases in snakebite risk (as measured by VOI) are concentrated in regions with high population density but low levels of urbanization, a demographic pattern typical of many developing countries. These areas often lack adequate health infrastructure and access to timely clinical care, making them more vulnerable to the growing burden of envenomation (Harrison et al. 2009). Given the spatial heterogeneity and anticipated shifts in snake distributions, there is an urgent need for high-resolution, regionally tailored risk maps that integrate climate projections, species co-occurrence data, and human exposure patterns. These forecasts can inform antivenom stockpiling and distribution strategies, ensuring timely access to treatment in newly affected areas, mainly in resource-limited settings where snakebite mortality remains high. To effectively reduce mortality and improve outcomes in regions with rising VOI and limited infrastructure, public health systems should prioritize the development of polyvalent antivenoms that can neutralize both neurotoxic and hemotoxic species, and the establishment of regional venom banks to secure reliable supplies for antivenom production. These efforts should be supported by the creation of high-resolution, dynamic risk maps integrating real-time environmental and demographic data, which can guide strategic stockpiling and timely distribution of antivenoms in newly affected areas. In some regions, such as parts of sub-Saharan Africa, temporary declines in venomous snake diversity may simplify species identification and facilitate clinical management. Ongoing climate and land-use changes are expected to alter local snake communities, introducing new venom profiles and complicating clinical management. To mitigate these emerging challenges, prevention strategies should incorporate continuous ecological monitoring to reduce snakebite-related morbidity and mortality in a rapidly changing world.

## Limitations

This study is subject to several important limitations. First, our models do not account for interspecies variation in venom potency, such as LD₅₀ values, which may affect clinical severity. Second, we assume static land-use and human exposure patterns, whereas future changes in urbanization, agriculture, or behavior could dramatically shift exposure dynamics. Third, the models do not incorporate healthcare system changes (e.g., access to hospitals, antivenom coverage), which will also mediate future snakebite burden. Lastly, significant data gaps in Southeast Asia and Central Africa may lead to regional underestimation of risk. Although we found limited future increases in VRI across Southeast Asia, this may reflect data scarcity rather than true ecological patterns; the current projections may underestimate actual snakebite risk in these areas. Future work should aim to integrate toxin-specific pharmacological data, adaptive healthcare scenarios, and improved species occurrence data to refine projections.

## Supplementary Material

zoaf070_Supplementary_Data

## Data Availability

Datasets supporting the findings of this study, including species occurrence records and species trait information, are publicly available in the Figshare repository at https://doi.org/10.6084/m9.figshare.30509042.
