# Supplementary material for: Global venomous snake redistribution and snakebite risk under climate change
Source: Curr Zool. 2025 Nov 6;72(4):579–89. doi: 10.1093/cz/zoaf070 (PMC13500158; doi:10.1093/cz/zoaf070)
Supplement: Supplementary file 1 [file zoaf070_supplementary_data.docx]

**Supporting information**

**Table S1 Taxonomic summary of 373 medically important snake species recognized by the WHO**. Species were summarized by venom type (neurotoxic, hemotoxic, both, none, or unknown) and WHO category (category 1, category 2, or both). "Unknown" refers to species lacking recorded information on venom toxicity.

| Group | Neurotoxic | Hemotoxic | Both | None | Unknown | Total |
| --- | --- | --- | --- | --- | --- | --- |
| Category 1 | 48 | 59 | 11 | 12 | 22 | 130 |
| Category 2 | 88 | 96 | 14 | 93 | 58 | 321 |
| Both Categories | 31 | 34 | 5 | 7 | 11 | 78 |
| Total | 105 | 121 | 20 | 98 | 69 | 373 |

**Table S2 Taxonomic summary of 273 medically important snake species with >5 valid occurrence records in this study**. Species were summarized by venom type (neurotoxic, hemotoxic, both, none, or unknown) and WHO category (category 1, category 2, or both).

| Group | Neurotoxic | Hemotoxic | Both | None | Total |
| --- | --- | --- | --- | --- | --- |
| Category1 | 43 | 54 | 10 | 12 | 99 |
| Category2 | 82 | 86 | 14 | 83 | 237 |
| Both Categories | 28 | 33 | 5 | 7 | 63 |
| Total | 97 | 107 | 19 | 88 | 273 |

**Table S3. List of environmental and anthropogenic variables used in species distribution models.** All variables were resampled to the same resolution of 0.25° (~27 km) using bilinear interpolation (3 × 3 cell window) for model analysis.

| **Variable Name** | **Description** | **Source** | **Resolution** |
| --- | --- | --- | --- |
| Mean annual temperature | Annual Mean Temperature (Bio1) | WorldClim v2.1 (Fick & Hijmans) | 0.1667° (~18.5 km) |
| Annual precipitation | Annual Precipitation (Bio12) | WorldClim v2.1 (Fick & Hijmans) | 0.1667° (~18.5 km) |
| Precipitation seasonality | Precipitation Seasonality (Coefficient of Variation, Bio15) | WorldClim v2.1 (Fick & Hijmans) | 0.1667° (~18.5 km) |
| Primary forest | Fractional cover of primary forest | LUH2 (Chini et al. 2014) | 0.25° (~27 km) |
| Secondary forest | Fractional cover of secondary forest | LUH2 (Chini et al. 2014) | 0.25° (~27 km) |
| Rangeland | Fractional cover of natural grassland | LUH2 (Chini et al. 2014) | 0.25° (~27 km) |
| Pasture | Fractional cover of grazing land | LUH2 (Chini et al. 2014) | 0.25° (~27 km) |
| Primary non-forest | Fractional cover of non-forested primary land | LUH2 (Chini et al. 2014) | 0.25° (~27 km) |
| Secondary non-forest | Fractional cover of potentially non-forested secondary land | LUH2 (Chini et al. 2014) | 0.25° (~27 km) |
| Urban | Fractional cover of urban land | LUH2 (Chini et al. 2014) | 0.25° (~27 km) |
| Population density | Total projected population count per 1-km grid cell | (Gao 2017) | 0.0083° (~1 km) |
| Elevation | Average elevation above sea level | WorldClim v2.1 (Fick & Hijmans) | 0.1667° (~18.5 km) |

Note: All land-use variables are expressed as fractional cover (ranging from 0 to 1) per grid cell. Spatial resolutions are reported in decimal degrees with approximate linear distances at the equator.

**Table S4. Comparison of model parameter estimates with and without IUCN-based random points in Southeast Asia.** Posterior means and 95% credible intervals (2.5% and 97.5% quantiles) are shown for each predictor variable. Results indicate high consistency between the two approaches.

| Variable | Without Random Points | | | With Random Points | | |
| --- | --- | --- | --- | --- | --- | --- |
|  | Mean | 2.5% CI | 97.5% CI | Mean | 2.5% CI | 97.5% CI |
| Temperature | -7.214 | -7.564 | -6.863 | -5.717 | -6.016 | -5.419 |
| Precipitation | -1.372 | -1.673 | -1.07 | -0.524 | -0.785 | -0.264 |
| Seasonality | -1.901 | -2.394 | -1.407 | -2.032 | -2.407 | -1.657 |
| Primary forest | 0.903 | 0.841 | 0.964 | 0.922 | 0.861 | 0.983 |
| Secondary forest | -0.971 | -1.025 | -0.916 | -0.972 | -1.027 | -0.918 |
| Pasture | -0.326 | -0.366 | -0.287 | -0.333 | -0.372 | -0.293 |
| Rangeland | -0.876 | -0.941 | -0.811 | -0.951 | -1.016 | -0.886 |
| Primary non-forest | -0.653 | -0.714 | -0.592 | -0.681 | -0.742 | -0.62 |
| Secondary nonforest | -0.577 | -0.641 | -0.513 | -0.591 | -0.655 | -0.528 |
| Human population | 0.148 | 0.067 | 0.23 | 0.27 | 0.188 | 0.351 |
| Urban | -0.071 | -0.094 | -0.048 | -0.073 | -0.096 | -0.05 |
| Altitude | -0.48 | -0.564 | -0.396 | -0.472 | -0.553 | -0.391 |

**Table S5 List of 193 medically important snake species successfully modeled using Maxent in this study.**

| Family | Scientific name |
| --- | --- |
| Atractaspididae | *Atractaspis bibronii* |
| Atractaspididae | *Atractaspis engaddensis* |
| Atractaspididae | *Atractaspis fallax* |
| Atractaspididae | *Atractaspis microlepidota* |
| Atractaspididae | *Atractaspis micropholis* |
| Colubridae | *Dispholidus typus* |
| Colubridae | *Rhabdophis tigrinus* |
| Colubridae | *Thelotornis capensis* |
| Colubridae | *Thelotornis kirtlandii* |
| Colubridae | *Thelotornis mossambicanus* |
| Elapidae | *Acanthophis antarcticus* |
| Elapidae | *Acanthophis cryptamydros* |
| Elapidae | *Acanthophis hawkei* |
| Elapidae | *Acanthophis praelongus* |
| Elapidae | *Acanthophis pyrrhus* |
| Elapidae | *Acanthophis rugosus* |
| Elapidae | *Austrelaps labialis* |
| Elapidae | *Austrelaps ramsayi* |
| Elapidae | *Austrelaps superbus* |
| Elapidae | *Bungarus caeruleus* |
| Elapidae | *Bungarus candidus* |
| Elapidae | *Bungarus fasciatus* |
| Elapidae | *Bungarus multicinctus* |
| Elapidae | *Calliophis intestinalis* |
| Elapidae | *Dendroaspis angusticeps* |
| Elapidae | *Dendroaspis jamesoni* |
| Elapidae | *Dendroaspis polylepis* |
| Elapidae | *Dendroaspis viridis* |
| Elapidae | *Hemachatus haemachatus* |
| Elapidae | *Hoplocephalus bitorquatus* |
| Elapidae | *Hoplocephalus bungaroides* |
| Elapidae | *Hoplocephalus stephensii* |
| Elapidae | *Micruroides euryxanthus* |
| Elapidae | *Micrurus altirostris* |
| Elapidae | *Micrurus brasiliensis* |
| Elapidae | *Micrurus browni* |
| Elapidae | *Micrurus circinalis* |
| Elapidae | *Micrurus corallinus* |
| Elapidae | *Micrurus diastema* |
| Elapidae | *Micrurus elegans* |
| Elapidae | *Micrurus filiformis* |
| Elapidae | *Micrurus frontalis* |
| Elapidae | *Micrurus fulvius* |
| Elapidae | *Micrurus hemprichii* |
| Elapidae | *Micrurus ibiboboca* |
| Elapidae | *Micrurus isozonus* |
| Elapidae | *Micrurus laticollaris* |
| Elapidae | *Micrurus lemniscatus* |
| Elapidae | *Micrurus pyrrhocryptus* |
| Elapidae | *Micrurus spixii* |
| Elapidae | *Micrurus surinamensis* |
| Elapidae | *Micrurus tener* |
| Elapidae | *Naja anchietae* |
| Elapidae | *Naja annulata* |
| Elapidae | *Naja annulifera* |
| Elapidae | *Naja arabica* |
| Elapidae | *Naja ashei* |
| Elapidae | *Naja atra* |
| Elapidae | *Naja haje* |
| Elapidae | *Naja melanoleuca* |
| Elapidae | *Naja mossambica* |
| Elapidae | *Naja naja* |
| Elapidae | *Naja nigricincta* |
| Elapidae | *Naja nigricollis* |
| Elapidae | *Naja nivea* |
| Elapidae | *Naja nubiae* |
| Elapidae | *Naja oxiana* |
| Elapidae | *Naja pallida* |
| Elapidae | *Naja philippinensis* |
| Elapidae | *Naja samarensis* |
| Elapidae | *Naja sputatrix* |
| Elapidae | *Notechis scutatus* |
| Elapidae | *Ophiophagus hannah* |
| Elapidae | *Oxyuranus microlepidotus* |
| Elapidae | *Oxyuranus scutellatus* |
| Elapidae | *Oxyuranus temporalis* |
| Elapidae | *Pseudechis australis* |
| Elapidae | *Pseudechis butleri* |
| Elapidae | *Pseudechis colletti* |
| Elapidae | *Pseudechis guttatus* |
| Elapidae | *Pseudechis pailsei* |
| Elapidae | *Pseudechis porphyriacus* |
| Elapidae | *Pseudechis weigeli* |
| Elapidae | *Pseudohaje nigra* |
| Elapidae | *Pseudonaja affinis* |
| Elapidae | *Pseudonaja aspidorhyncha* |
| Elapidae | *Pseudonaja guttata* |
| Elapidae | *Pseudonaja inframacula* |
| Elapidae | *Pseudonaja ingrami* |
| Elapidae | *Pseudonaja mengdeni* |
| Elapidae | *Pseudonaja nuchalis* |
| Elapidae | *Pseudonaja textilis* |
| Elapidae | *Tropidechis carinatus* |
| Elapidae | *Walterinnesia aegyptia* |
| Viperidae | *Agkistrodon bilineatus* |
| Viperidae | *Agkistrodon contortrix* |
| Viperidae | *Agkistrodon piscivorus* |
| Viperidae | *Agkistrodon taylori* |
| Viperidae | *Bitis arietans* |
| Viperidae | *Bitis gabonica* |
| Viperidae | *Bitis rhinoceros* |
| Viperidae | *Bothriechis marchi* |
| Viperidae | *Bothriechis schlegelii* |
| Viperidae | *Bothrocophias hyoprora* |
| Viperidae | *Bothrops alternatus* |
| Viperidae | *Bothrops ammodytoides* |
| Viperidae | *Bothrops atrox* |
| Viperidae | *Bothrops brazili* |
| Viperidae | *Bothrops diporus* |
| Viperidae | *Bothrops erythromelas* |
| Viperidae | *Bothrops jararaca* |
| Viperidae | *Bothrops jararacussu* |
| Viperidae | *Bothrops jonathani* |
| Viperidae | *Bothrops leucurus* |
| Viperidae | *Bothrops lutzi* |
| Viperidae | *Bothrops marmoratus* |
| Viperidae | *Bothrops moojeni* |
| Viperidae | *Bothrops neuwiedi* |
| Viperidae | *Bothrops pauloensis* |
| Viperidae | *Calloselasma rhodostoma* |
| Viperidae | *Cerastes cerastes* |
| Viperidae | *Cerastes gasperettii* |
| Viperidae | *Cerrophidion godmani* |
| Viperidae | *Crotalus adamanteus* |
| Viperidae | *Crotalus atrox* |
| Viperidae | *Crotalus basiliscus* |
| Viperidae | *Crotalus cerastes* |
| Viperidae | *Crotalus culminatus* |
| Viperidae | *Crotalus durissus* |
| Viperidae | *Crotalus enyo* |
| Viperidae | *Crotalus horridus* |
| Viperidae | *Crotalus intermedius* |
| Viperidae | *Crotalus lepidus* |
| Viperidae | *Crotalus mitchellii* |
| Viperidae | *Crotalus molossus* |
| Viperidae | *Crotalus oreganus* |
| Viperidae | *Crotalus ornatus* |
| Viperidae | *Crotalus pricei* |
| Viperidae | *Crotalus ravus* |
| Viperidae | *Crotalus ruber* |
| Viperidae | *Crotalus scutulatus* |
| Viperidae | *Crotalus simus* |
| Viperidae | *Crotalus totonacus* |
| Viperidae | *Crotalus triseriatus* |
| Viperidae | *Crotalus tzabcan* |
| Viperidae | *Crotalus viridis* |
| Viperidae | *Crotalus willardi* |
| Viperidae | *Daboia palaestinae* |
| Viperidae | *Daboia russelii* |
| Viperidae | *Daboia siamensis* |
| Viperidae | *Deinagkistrodon acutus* |
| Viperidae | *Echis carinatus* |
| Viperidae | *Echis coloratus* |
| Viperidae | *Echis leucogaster* |
| Viperidae | *Echis ocellatus* |
| Viperidae | *Echis pyramidum* |
| Viperidae | *Gloydius halys* |
| Viperidae | *Gloydius intermedius* |
| Viperidae | *Gloydius ussuriensis* |
| Viperidae | *Lachesis muta* |
| Viperidae | *Montivipera bornmuelleri* |
| Viperidae | *Montivipera raddei* |
| Viperidae | *Montivipera xanthina* |
| Viperidae | *Ophryacus undulatus* |
| Viperidae | *Porthidium nasutum* |
| Viperidae | *Porthidium ophryomegas* |
| Viperidae | *Porthidium yucatanicum* |
| Viperidae | *Protobothrops flavoviridis* |
| Viperidae | *Protobothrops jerdonii* |
| Viperidae | *Protobothrops mucrosquamatus* |
| Viperidae | *Pseudocerastes persicus* |
| Viperidae | *Sistrurus catenatus* |
| Viperidae | *Sistrurus miliarius* |
| Viperidae | *Trimeresurus albolabris* |
| Viperidae | *Trimeresurus erythrurus* |
| Viperidae | *Trimeresurus flavomaculatus* |
| Viperidae | *Trimeresurus gumprechti* |
| Viperidae | *Trimeresurus insularis* |
| Viperidae | *Trimeresurus macrops* |
| Viperidae | *Trimeresurus purpureomaculatus* |
| Viperidae | *Trimeresurus stejnegeri* |
| Viperidae | *Trimeresurus venustus* |
| Viperidae | *Trimeresurus vogeli* |
| Viperidae | *Trimeresurus yunnanensis* |
| Viperidae | *Vipera ammodytes* |
| Viperidae | *Vipera aspis* |
| Viperidae | *Vipera berus* |
| Viperidae | *Vipera eriwanensis* |
| Viperidae | *Vipera latastei* |
| Viperidae | *Vipera monticola* |
| Viperidae | *Vipera renardi* |
| Viperidae | *Vipera seoanei* |
| Viperidae | *Vipera ursinii* |

**Table S6 Taxonomic summary of 193 medically important snake species successfully modeled using Maxent in this study**. Species were summarized by venom type (neurotoxic, hemotoxic, both, none, or unknown) and WHO category (category 1, category 2, or both).

| Group | Neurotoxic | Hemotoxic | Both | None | Total |
| --- | --- | --- | --- | --- | --- |
| Category1 | 36 | 48 | 10 | 9 | 83 |
| Category2 | 60 | 66 | 12 | 52 | 166 |
| Both Categories | 23 | 32 | 5 | 6 | 56 |
| Total | 73 | 82 | 17 | 55 | 193 |

**Table S7 Taxonomic summary of the 193 medically important snake species successfully modeled using Maxent in this study, categorized by family**. Species were summarized by venom type (neurotoxic, hemotoxic, both, none, or unknown) and WHO category (category 1, category 2, or both).

| Family | Number of  Genera | Number of Species | | | | |
| --- | --- | --- | --- | --- | --- | --- |
|  |  | Neurotoxic | Hemotoxic | Both | None | Total |
| Atractaspididae | 1 | 0 | 0 | 0 | 5 | 5 |
| Colubridae | 3 | 0 | 5 | 0 | 0 | 5 |
| Elapidae | 18 | 65 | 19 | 13 | 13 | 84 |
| Viperidae | 22 | 8 | 58 | 4 | 37 | 99 |
| Total | 44 | 73 | 82 | 17 | 55 | 193 |

**Figure S1 Spatial distribution of total occurrences (A) and species richness (B).** Warmer colors indicate higher sample size or species richness, while cooler colors represent lower values.

**Figure S2 Projected global category 1 venomous snake species richness across three time periods (2041-2060, 2061-2080, 2081-2100) under four different SSP climate scenarios (SSP126, SSP245, SSP370, SSP585).** Warmer colors indicate higher snake richness, while cooler colors represent lower richness.

**Figure S3. Temporal changes in the Snakebite Range (SR) for hemotoxic snake species under different climate change scenarios and VRI thresholds.** Each panel illustrates the number of grid cells exceeding a given VRI threshold (4 - 12 species) across four future time intervals (2021–2040, 2041–2060, 2061–2080, and 2081–2100) under four SSP scenarios (SSP126, SSP245, SSP370, SSP585).

**Figure S4 Temporal changes in the snakebite range (SR) for category 1 (A-D), category 2 (E-H), neurotoxic (I-L), and hemotoxic (M-P) snake species across four biogeographic regions (tropical, arid, temperate, cold) under climate change scenarios.** The SR was measured by the number of grids with VRI greater than 5. Lines and points represent the mean number of grids across 10 climate models, with error bars indicating standard deviation. Global-scale changes were represented in Fig. 2.

**Figure S5 SREI (Snakebite Range Expansion Index) across tropical (A), cold (B), arid (C), and temperate (D) regions.** Points represent SREI, with 95% CI. Points outside the dashed zero line indicate range expansion, while points inside indicate contraction. Colors denote toxin type classification: hemotoxic (red), neurotoxic (green), both (purple), and neither (blue), and the inset pie chart denotes the number of species per toxin type. The corresponding pooled global trend was shown in Fig. 3A.

**Figure S6 Temporal trend in the correlations between neurotoxic VRI and hemotoxic VRI in tropical (A), arid (B), temperate (C), and cold (D) regions under climate change.** Spearman’s correlation coefficients were averaged across climate models per scenario and time period, with error bars showing standard deviation. The corresponding pooled global trend was shown in Fig. 3B.
